# Three-dimensional and artificial intelligence-assisted preoperative templating in total hip arthroplasty: a systematic review and meta-analysis

**DOI:** 10.1186/s42836-026-00417-3

**Published:** 2026-08-04

**Authors:** Seyed Pouya Taghavi, Ali Salmani, Taner Karlidag, Aziz Emre Nokay, Sukrit J. Suresh, Janet D. Conway, Amir Human Hoveidaei, Mustafa Citak

**Affiliations:** 1 Neuromusculoskeletal Research Center, Iran University of Medical Sciences, Tehran, 1449614535 Iran; 2 Department of Orthopaedics and Traumatology, Gaziantep City Hospital, Gaziantep, 27470 Türkiye; 3 Department of Trauma Surgery, University Medical Center Regensburg, Regensburg, 93053 Germany; 4 Department of Orthopaedic Surgery, Johns Hopkins University, Baltimore, MD 21287 USA; 5 International Center for Limb Lengthening, Rubin Institute for Advanced Orthopedics, Sinai Hospital of Baltimore, Baltimore, MD 21215 USA; 6 Knappschaft Kliniken Dortmund, Brackel, 44309 Germany

**Keywords:** Total hip arthroplasty, Preoperative templating, Three-dimensional planning, Artificial intelligence, Implant sizing accuracy

## Abstract

**Background:**

Preoperative templating in total hip arthroplasty (THA) optimizes implant sizing and positioning accuracy. Standard practice relies on two-dimensional (2D) templating, while three-dimensional (3D) and AI-assisted planning are evolving alternatives. This study aimed to evaluate the implant size prediction accuracy and its impact on intraoperative and postoperative outcomes.

**Methods:**

We conducted a systematic review and meta-analysis in April 2025 across MEDLINE (PubMed), Scopus, and Web of Science to identify studies comparing advanced preoperative planning (AI-based or 3D) with 2D planning in THA. Data on femoral stem and acetabular cup sizing accuracy and key clinical outcomes were extracted. Study quality was evaluated using the Joanna Briggs Institute (JBI) critical appraisal checklist. Overall, 32 studies were identified.

**Results:**

Meta-analysis showed that 3D planning had significantly higher exact implant size agreement than 2D (femoral OR = 2.00, 95% CI 1.34–2.97; acetabular OR = 5.32, 95% CI 1.89–14.94), and significantly lower major deviation (femoral OR = 0.37, 95% CI 0.24–0.58; acetabular OR = 0.18, 95% CI 0.06–0.50). AI-assisted planning showed significantly higher exact implant size agreement (femoral OR = 3.27, 95% CI 2.52–4.24; acetabular OR = 3.96, 95% CI 2.95–5.32) and significantly lower major deviation (femoral OR = 0.22, 95% CI 0.16–0.31; acetabular OR = 0.29, 95% CI 0.15–0.56). Significantly reduced blood loss (MD = − 36.21, 95% CI − 46.58 to − 25.85), operative time (MD = − 15.54, 95% CI − 24.25 to − 6.83), LLD (MD = − 1.58, 95% CI − 2.22 to − 0.93), and higher abduction angle (MD: 1.64; 95% CI 0.37–2.92) were associated with AI-based planning. Postoperative Harris Hip Score was marginally higher with AI-assisted planning (MD = 0.73 points), but this difference was below the minimal clinically important threshold and not robust to sensitivity analysis.

**Conclusion:**

Advanced planning improved implant sizing accuracy. AI-assisted planning was additionally associated with more favorable intraoperative and postoperative outcomes; however, heterogeneity, limited long-term data, and restricted external validity due to the geographic concentration of AI-assisted studies and limited diversity of AI software platforms warrant further standardized comparative studies.

**Supplementary Information:**

The online version contains supplementary material available at 10.1186/s42836-026-00417-3.

## Introduction

Preoperative planning is crucial to successful total hip arthroplasty (THA). It defines implant size and position, improves prediction of limb length and offset, restores biomechanics, reduces outliers in component alignment, and supports minimally invasive approaches. Inaccurate planning can lead to instability and dislocation, which may increase the risk of complications, revision surgery, and patient dissatisfaction [1–3]. Surgeons commonly use two-dimensional (2D) radiographs or three-dimensional (3D) imaging modalities, including computed tomography (CT) or low-dose EOS imaging, to select implants, anticipate surgical difficulties, and address intraoperative uncertainty [2, 4–6]. More recently, advanced planning approaches (3D and artificial intelligence (AI)-assisted) have demonstrated greater accuracy in predicting implant size and early functional outcomes than conventional 2D templating, although these approaches may involve higher costs and additional radiation exposure [1, 6–8].

In hip arthroplasty, prior evidence suggested that 3D planning improved implant size prediction, but its impact on clinical outcomes and long-term implant survival has not been systematically evaluated [9]. In addition, the THA literature has largely focused on comparisons between 2 and 3D templating modalities, leaving AI-driven planning models undercharacterized. Consequently, while 3D planning may be beneficial, its routine use in THA is not universally endorsed, and further research is needed to establish standardized guidance and demonstrate cost-effectiveness.

This study aims to compare 3D and AI-assisted preoperative planning with conventional 2D templating in THA through a systematic review and meta-analysis. Specifically, we sought to evaluate: (1) femoral stem sizing accuracy for 3D and AI-assisted planning versus 2D planning, using common criteria such as exact size agreement, agreement within one size, and more than two component sizes agreement; (2) acetabular cup sizing accuracy using the same criteria; and (3) extent to which differences in planning accuracy are reflected in intraoperative and postoperative outcomes. By doing so, we aim to provide a clearer understanding of the potential advantages and current limitations of 3D and AI-based planning in THA.

## Methods

This systematic review and meta-analysis were conducted in accordance with the Preferred Reporting Items for Systematic Reviews and Meta-Analyses (PRISMA) 2020 guidelines [10, 11]. The study protocol was prospectively registered in the International Prospective Register of Systematic Reviews (CRD420251270329).

### Search strategy

A comprehensive literature search was performed on April 24, 2025, across MEDLINE (PubMed), Scopus, and Web of Science using keywords including “Arthroplasty, Replacement, Hip,” “Preoperative Care,” “Patient Care Planning,” and “Acetabulum/surgery” (full search strategy in Supplementary Table S2). No restrictions were applied for publication date or study location. To ensure completeness, reference lists of included studies and articles citing them were manually screened to identify additional relevant studies. All citations were imported into Rayyan, a web-based platform for systematic review screening, and duplicates were removed using reference management software (EndNote) [12]. Titles and abstracts were independently screened by two reviewers (EN, TK), followed by full-text review. Discrepancies were resolved through discussion with a third reviewer.

### Eligibility criteria

Eligible studies included adult patients undergoing primary or revision THA in which 3D or AI-assisted preoperative planning was evaluated. Comparative studies directly comparing 3D or AI-assisted planning with conventional 2D planning were included in the quantitative meta-analysis when extractable outcome data were available. Single-arm studies of 3D or AI-assisted planning were included only for descriptive synthesis of planning accuracy and did not contribute to pooled comparative estimates. Exclusion criteria encompassed non-English publications, case reports, reviews, conference abstracts, or studies lacking a comparator group or relevant outcome data. Outcomes of interest included measures of femoral stem and acetabular cup sizing accuracy, implant positioning, leg-length discrepancy (LLD), hip functional scores such as the Harris Hip Score (HHS), operative time, intraoperative blood loss, and other relevant perioperative or postoperative parameters. This comprehensive approach ensured inclusion of studies with clinically meaningful and quantifiable endpoints relevant to THA planning accuracy and surgical outcomes.

### Data extraction

From the eligible studies, two reviewers (EN, TK) independently extracted data using preformatted Microsoft Excel spreadsheets. Extracted information included study characteristics (title, first author, publication year, study design), participant demographics (age, sex, sample size), type of intervention, and inclusion criteria. Reported outcomes encompassed femoral stem and acetabular cup sizing accuracy, HHS, LLD, abduction angle, operative time, and intraoperative blood loss. Any disagreements during data extraction were resolved through discussion with a third reviewer to ensure accuracy and consistency. This structured extraction allowed for uniform analysis across studies and facilitated reliable synthesis of surgical and clinical outcomes associated with different preoperative planning strategies.

### Risk of bias assessment

The Joanna Briggs Institute (JBI) critical appraisal checklists were used to evaluate the quality of the included studies [13]. Two reviewers (EN, TK) independently assessed each study based on its design, and discrepancies were resolved through discussion. The checklists assessed various domains, such as inclusion criteria, group comparability and confounding identification, valid and reliable measurement of outcomes, adequacy and completeness of follow-up, and suitability of statistical analysis, by rating each question yes, no, unclear, or not applicable.

Overall, all cross-sectional studies and all case series met JBI items. In cohort studies, outcome measurement and statistical analysis were generally adequate. However, the most frequent limitations are related to insufficient reporting of confounding and incomplete or unclear follow-up. Among randomized controlled trials (RCTs), randomization and baseline comparability were commonly reported. In contrast, allocation concealment and blinding were often unclear or not feasible, with some concerns also noted for follow-up adequacy. The single case–control study had limited reporting on confounding identification and control (see supplementary material).

### Data analysis

All statistical analyses were conducted using Review Manager (RevMan, Version 5.4, Cochrane Collaboration, 2020) [14]. Dichotomous outcomes were analyzed using odds ratios (ORs) with 95% confidence intervals (CIs), while continuous outcomes were assessed using mean differences (MDs). Heterogeneity among included studies was evaluated using the *Chi*-square (χ^2^) test with a significance threshold of *p-*values < 0.05 and quantified with the I^2^ statistic. Heterogeneity levels were interpreted as low (I^2^ = 25 to 49%), moderate (I^2^ = 50 to 74%), or substantial (I^2^ ≥ 75%). Given the expected clinical and methodological heterogeneity among studies, a random-effects model was used for all analyses to account for between-study variability and provide more conservative, generalizable estimates. Additionally, “Leave-one-out” sensitivity analyses were performed for clinically key outcomes with substantial heterogeneity by omitting one individual study at a time to assess the robustness of pooled estimates. Publication bias was evaluated for outcomes involving at least 10 studies through visual inspection of funnel plots (RevMan v5.4.1) (Fig. 5) and quantitative validation via Egger’s regression test (Python statsmodels) (see supplementary material).

### Demographics and study selection

**Fig. 1:**
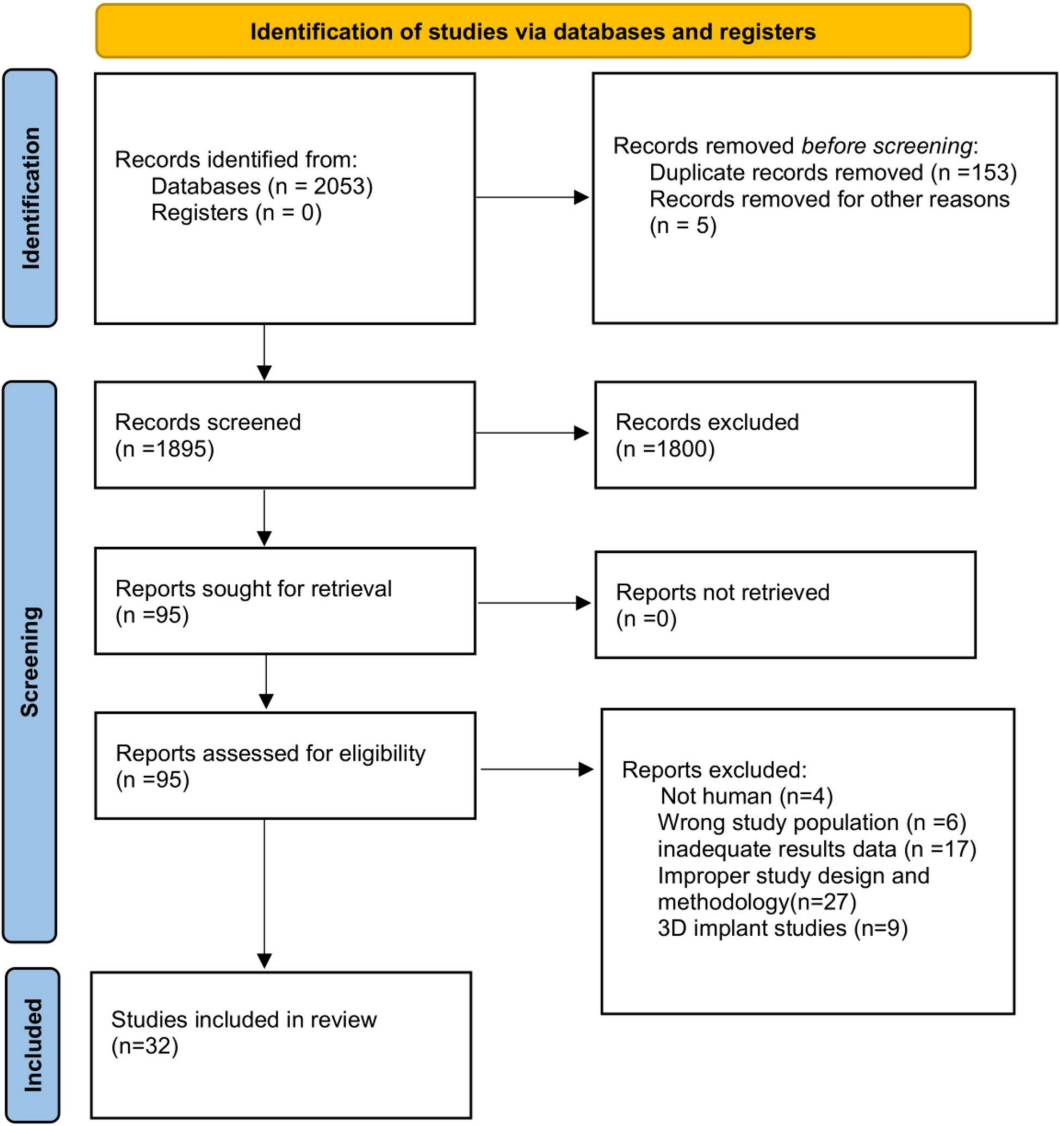
PRISMA 2020 flow diagram of the systematic study selection process

A total of 2,053 studies were identified through electronic searches of PubMed/MEDLINE, Scopus, and Web of Science. The selection process is displayed in Fig. 1 [11]. After removing 153 duplicates via EndNote’s deduplication tool, 1,895 records underwent title and abstract screening, of which 1,855 were excluded. Following full-text assessment, 32 unique studies were included [1–4, 6, 7, 15–40]. Of these, 17 studies evaluated 3D planning, 14 evaluated AI-assisted planning, and one study contributed data to both planning categories. Therefore, the 32 unique studies yielded 33 planning-category records. Comparative data versus 2D planning were extracted where available, while single-arm 3D studies were included only for descriptive synthesis of planning accuracy. The included studies comprised 17 cohort studies, 5 randomized controlled trials, 4 cross-sectional analyses, 5 case series, and 1 case–control study.

In the first group (3D vs 2D), six studies originated from France, Four from China, two from Japan, two from Germany, and one each from the United States, Italy, the United Kingdom, Spain, and Switzerland. Publications spanned from 2003 to 2024, including a total of 1,583 patients, with a mean age ranging from 45 to 74 years. The 3D planning tools used were HipPlan software (four studies), EOS (three), ZedHip (two), Mimics (Three), Stryker (two), Patient-specific models (one), Hip-Op (one), robotic-assisted planning software (one), and OPSInsight (one).

In the second group (AI vs 2D), all studies were conducted in China, published between 2021 and 2025, and included 1,889 patients, with a mean age between 42.3 and 64.2 years. AI-based planning tools comprised AIHIP (13 studies), computer-aided design simulation (one), and robotic-assisted software (one). Study characteristics are summarized in Table 1.

**Table 1:** Study characteristics by planning category

Author	Year	Region	Design	N Hips(W/M)	Mean Age	Mean BMI	Mean F-U (m)	Planning platform
**3D planning**								
Mainard et al. [7]	2017	France	Case- Control	31 (21/10)	66	-	-	EOS
Tostain et al. [15]	2019	France	Retrospective single-arm cohort	61 (45/16)	74	30.5	10	HipPlan
Vaquinhas et al. [16]	2022	Spain	Historical-control cohort study	45 (27/18)	71.6	30.7	32.4	Patient-specific printed model
Brenneis et al. [17]	2020	Germany	RCT	51 (25/26)	60.2	27.8	-	EOS
Knafo et al. [2]	2019	France	Case series	33 (19/14)	65	27	-	EOS
Hassani et al. [1]	2014	Switzerland	Case series	50 (30/20)	64	-	-	HipPlan
Inoue et al. [18]	2015	Japan	Case series	65 (57/5)	60.3	-	0.5	ZedHip
Fontalis et al. [19]	2024	UK	RCT	60 (30/30)	65	26.7	-	Robotic arm-assisted
Aubert et al. [4]	2023	France	Retrospective comparative cohort	200 (111/89)	64.5	21.95	-	OPSInsight
Schiffner et al. [20]	2018	Germany	Cross-sectional	116 (40/76)	69.2	-	-	ZedHip
Crutcher et al. [21]	2023	USA	Cross-sectional	290	-	-	-	Stryker
Viceconti et al. [22]	2003	Italy	Cross-sectional	29 (6/23)	48.4	-	-	Hip-Op
Sariali et al. [3]	2012	France	RCT	60 (16/44)	58.6	26.4	-	HipPlan
Sariali et al. [23]	2009	France	Case series	223 (136/87)	70	28	-	HipPlan
Ogawa et al. [24]	2017	Japan	Retrospective single-arm cohort study	124 (11/113)	60.0	23.2	-	Stryker
Wu et al. [25]	2018	China	Case series	49	58.33	-	32.4	Mimics
Zeng et al. [26]	2014	China	Prospective comparative cohort	20 (4/16)	45	24	-	Mimics
Hou et al. [27]*	2021	China	Cross-sectional	59 (24/29)	57.4	-	-	Mimics, AIHIP
**AI-assisted planning**								
Anwar et al. [28]	2024	China	Prospective comparative cohort	117 (65/52)	62.3	25.5	-	AIHIP
Ding et al. [31]	2021	China	Retrospective comparative cohort	316 (124/192)	50.68	25.07	1.5	AIHIP
Lu et al. [32]	2025	China	Retrospective comparative cohort	96 (48/44)	64.22	23.70	24	AIHIP
Wu et al. [33]	2023	China	Retrospective comparative cohort	61 (31/30)	59.2	25.99	12	AIHIP
Zheng et al. [40]	2025	China	Prospective comparative cohort	56 (30/26)	59.6	-	6	AIHIP
Xie et al. [35]	2024	China	Retrospective comparative cohort	103 (16/87)	42.3	24.0	-	AIHIP
Yang et al. [36]	2023	China	Retrospective comparative cohort	440 (193/247)	57.0	23.61	1	AIHIP
Li et al. [6]	2025	China	Retrospective comparative cohort	109 (51/58)	62.47	23.12	12	AI-HIP
Cheng et al. [30]	2024	China	Prospective comparative cohort	62 (30/32)	60.35	-	6	CAD simulative
Chen et al. [29]	2022	China	Prospective comparative cohort	120 (59/61)	50.69	25.14	-	AIHIP
Wu et al. [34]	2023	China	Retrospective comparative cohort	161 (101/60)	57.6	25.06	-	AIHIP
Zhang et al. [38]	2023	China	Retrospective comparative cohort	84 (48/19)	45.5	-	24.9	Robot-assisted
Zhang et al. [37]	2024	China	RCT	45 (24/21)	62.33	25.66	12	AIHIP
Zheng et al. [39]	2024	China	RCT	60	62.85	24.1	-	AIHIP
Hou et al. [27]*	2021	China	Cross-sectional	59 (24/29)	57.4 ± 1.7	-	-	AIHIP

*3D* three-dimensional, *2D* two-dimensional, *AI* artificial intelligence, *F-U* follow-up, *m* month, *RCT* randomized controlled trials, *N* number, *EOS* biplanar low-dose stereoradiography system, *CAD* computer-aided design, *W* women, *M* men

^*^ This study contributed data to both the 3D versus 2D and AI-assisted versus 2D comparisons. Therefore, 32 unique studies contributed 33 comparison-level records

## Results

### Femoral stem size accuracy

#### Three-dimensional versus two-dimensional

**Fig. 2:**
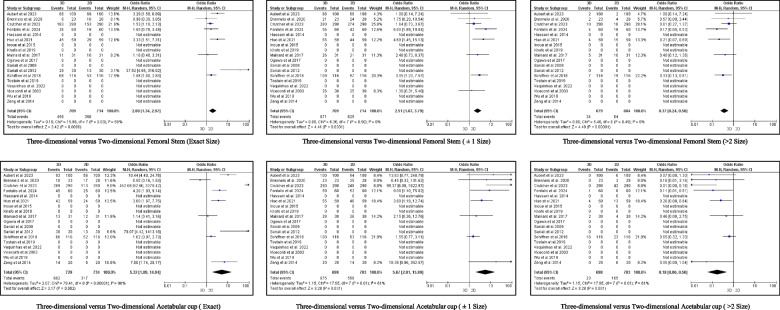
Forest plots comparing the accuracy of femoral stem and acetabular cup size prediction between three-dimensional (3D) and two-dimensional (2D) methods across accuracy categories (exact match, within one size, and > 2 sizes)

The pooled analysis demonstrated significantly higher femoral stem sizing accuracy for exact size agreement in 3D planning compared with 2D planning (OR = 2.00, 95% CI 1.34–2.97, *p*-value < 0.001; I^2^ = 56%) (Fig. 2). Accuracy was also significantly higher for agreement within one size (OR = 2.51, 95% CI 1.67–3.79, *p*-value < 0.001; I^2^ = 0%) (Fig. 2). The odds of major sizing deviation (an absolute discrepancy of > 2) were significantly lower with 3D planning relative to 2D templating (OR = 0.37, 95% CI 0.24–0.58, *p*-value < 0.001; I^2^ = 0%) (Fig. 2).

#### Artificial intelligence-assisted versus two-dimensional

**Fig. 3:**
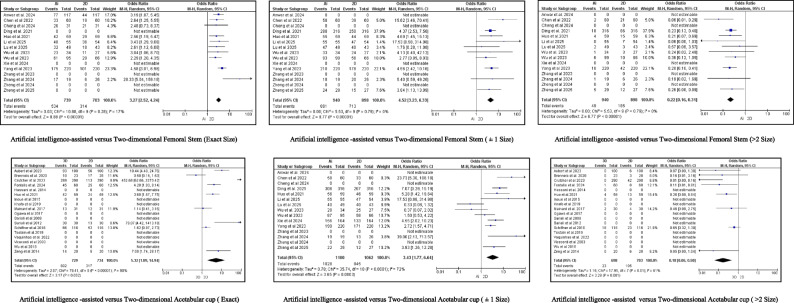
Forest plots comparing the accuracy of femoral stem and acetabular cup size prediction between artificial intelligence (AI)–assisted and two-dimensional (2D) methods across accuracy categories (exact match, within one size, and > 2 sizes)

**Fig. 4:**
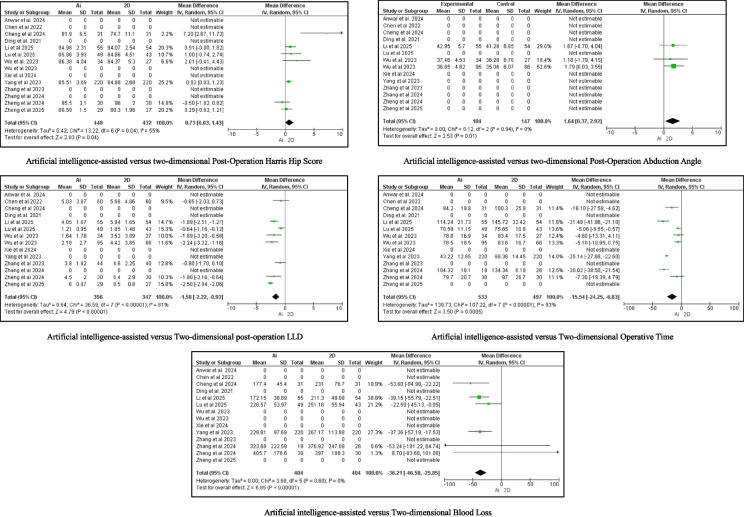
Funnel plots for publication bias assessment of artificial intelligence (AI)–assisted versus two-dimensional (2D) methods in femoral stem and acetabular cup size prediction categories

The meta-analysis showed that exact femoral stem size prediction was significantly higher in AI-assisted planning compared with 2D planning (OR = 3.27, 95% CI 2.52–4.24, *p*-value < 0.001; I^2^ = 17%) (Fig. 3). Accuracy was also significantly higher for agreement within one size with AI-assisted planning (OR = 4.52, 95% CI 3.23–6.33, *p-*value < 0.001; I^2^ = 0%) (Fig. 3), and the odds of major sizing deviation (an absolute discrepancy of > 2) were significantly lower (OR = 0.22, 95% CI 0.16–0.31, *p*-value < 0.001; I^2^ = 0%) (Fig. 3). However, funnel-plot asymmetry, supported by Egger’s test, suggested significant small-study effects for the major femoral stem deviation outcome (*p* = 0.002) (Fig. 4; see supplementary material).

### Acetabular cup size accuracy

#### Three-dimensional versus two-dimensional

The pooled analysis reported significantly higher odds of exact acetabular cup size prediction with 3D planning compared with 2D planning (OR = 5.32, 95% CI 1.89–14.94, *p*-value = 0.002; I^2^ = 90%) (Fig. 2). Leave-one-out analyses showed that the pooled effect for exact acetabular cup size agreement remained statistically significant. However, heterogeneity remained substantial across analyses. Accuracy was also significantly higher for prediction within one size with the 3D templating (OR = 5.67, 95% CI 2.01–15.99, *p*-value = 0.001; I^2^ = 61%) (Fig. 2). The odds of major sizing deviation (an absolute discrepancy of > 2) were significantly lower with 3D planning (OR = 0.18, 95% CI 0.06–0.50, *p*-value = 0.001; I^2^ = 61%) (Fig. 2).

#### Artificial intelligence-assisted versus two-dimensional

Exact acetabular cup size prediction was significantly more accurate with AI-based planning compared with 2D planning (OR = 3.96, 95% CI 2.95–5.32, *p*-value < 0.001; I^2^ = 41%) (Fig. 3). Pooled analysis also revealed significantly higher accuracy for agreement within one size with AI-assisted templating (OR = 3.43, 95% CI 1.77–6.64, *p*-value < 0.001; I^2^ = 72%) and the odds of major sizing deviation (an absolute discrepancy of > 2) were significantly lower (OR = 0.29, 95% CI 0.15–0.56, *p*-value < 0.001; I^2^ = 72%) (Fig. 3). Funnel-plot asymmetry and Egger’s test suggested significant small-study effects for the major acetabular cup deviation outcome (*p* = 0.002) (Fig. 4; see supplementary material). Stem and cup size prediction accuracy is summarized in Table 2.

**Table 2:** Accuracy of femoral stem and acetabular cup size prediction

Author	Indication for THA	Planning Method	N	Femoral Stem Type	Femoral Stem Size			Acetabular Cup Type	Acetabular Cup Size		
					Exact	Within ± 1 size	> ± 2 sizes		Exact	Within ± 1 size	> ± 2 sizes
Mainard et al. [7]	primary OA	3D	31	Excia Hip Stem System (Aesculap), double taper wedge, cementless	34% (11/31)	84% (26/31)	16% (5/31)	Plasmacup SC (Aesculap), ceramic liner	41% (13/31)	93% (28/30)	7% (2/30)
		2D	31		32% (10/31)	68% (21/31)	32% (10/31)		40% (12/31)	87% (26/30)	13% (4/30)
Brenneis et al. [17]	primary OA	3D	23	Alloclassic Zweymüller SL/SLO (cementless, press-fit) and Optimys short stem (cementless, metaphyseal-anchoring)	34.8% (8/23)	91.3% (21/23)	8.6% (2/23)	Cementless press-fit Allofit cup (Zimmer Inc.)	43.5% (10/23)	100% (23/23)	0% (0/23)
		2D	28		35.7% (10/28)	85.7% (24/28)	14.2% (4/28)		60.7% (17/28)	89.3% (25/28)	10.71% (3/28)
Knafo et al. [2]	primary OA	3D	33	Excia™ (BBraun), uncemented, design not specified	54.5% (18/33)	94% (31/33)	6.06% (2/33)	Plasmacup™ SC (BBraun), uncemented, impacted	48.48% (16/33)	100% (33/33)	0% (0/33)
		2D									
Hassani et al. [1]	NR	3D	50	SPS system, cementless (design not specified)	94% (47/50)			Not Reported	100% (50/50)		
		2D									
Inoue et al. [18]	secondary OA (DDH)	3D	65	APS Natural-Hip™ System (Zimmer), cementless, anatomical short stem (fit-and-fill)	65% (42/65)	98% (63/65)	3.07% (2/65)	Trilogy acetabular cup (Zimmer Inc.)	92% (62/65)	100% (65/65)	0% (0/65)
		2D									
Fontalis et al. [19]	primary OA	3D	60	Accolade II (Stryker), cementless, proximally coated	43.4% (26/60)	93.4% (56/60)	6.6% (4/60)	Trident Acetabular System (Stryker), porous acetabular shell	75% (45/60)	98.4% (59/60)	1.6% (1/60)
		2D	60		31.7% (19/60)	70% (42/60)	30% (18/60)		41.7% (25/60)	86.7% (52/60)	13.3% (8/60)
Sariali et al. [3]	primary OA	3D	30	SPS (Symbios), cementless, anatomic modular neck stem	96% (29/30)			Delta Ceramic-on-ceramic heads and liners (Ceramtec) — specific cup model not specified	100% (30/30)		
		2D	30	Global (Ceramconcept), cementless, straight quadrangular stem	43% (13/30)				43% (13/30)		
Sariali et al. [23]	primary OA	3D	223	SPS-Modular (Symbios), cementless, anatomical stem	94% (209/223)	100% (223/223)	0% (0/223)	Hemispherical acetabular component with two peripheral ridges (specific brand not specified in excerpt)	86% (192/223)	100% (223/223)	0% (0/223)
		2D									
Aubert et al. [4]	NR	3D	100	Straight quadrangular cementless stem (brand not specified)	69% (88/100)	98% (98/100)	2% (2/100)	Meije Dynacup (Corin, Cirencester, UK), ceramic-on-ceramic	93% (93/100)	100% (100/100)	0% (0/100)
		2D	100		88% (69/100)	98% (98/100)	2% (2/100)		56% (56/100)	94% (94/100)	6% (6/100)
Schiffner et al. [20]	primary OA	3D	116	Multiple stems: Fitmore (short), SPP II (anatomical), Alloclassic, Avenir, MIA, all cementless	58.6% (68/116)	94% (109/116)	6% (7/116)	Allofit pressfit cup (Zimmer, Neu-Ulm, Germany)	56.9% (66/116)	86.2% (100/116)	13.8% (16/116)
		2D	116		45.7% (53/116)	83.6% (97/116)	16.3% (19/116)		44.8% (52/116)	80.2% (93/116)	19.8% (23/116)
Crutcher et al. [21]	NR	3D	290	Accolade II (Stryker), design not specified in paper	63.1% (183/290)	96.4% (280/290)	3.6% (10/290)	Trident IIR (Stryker, Mahwah, New Jersey)	99.7% (289/290)	100% (290/290)	0% (0/290)
		2D	290		52.9% (153/290)	94.5% (274/290)	5.5% (16/290)		39% (113/290)	85.5% (248/290)	14.5% (42/290)
Viceconti et al. [22]	DDH/primary OA/post-traumatic OA/Secondary OA (Perthes)/revision for infection	3D	30	AnCAFit, cementless, anatomic stem		86.2% (26/30)		Titanium alloy hemisphere covered with titanium alloy spheres and double layer of hydroxyapatite (AnCAFit system)			
		2D	30			82.7% (25/30)					
Ogawa et al. [24]	DDH/ON/primary OA/RDC	3D	124	CentPillar (Stryker), cementless, anatomical stem	86% (106/124)	100% (124/124)		Trident PSL cups (Stryker), cementless, titanium alloy with HA coating, 1.8 mm peripheral press fit	94% (116/124)	100% (124/124)	
		2D									
Wu et al. [25]	DDH	3D	49	Not Reported				Pinnacle (Depuy, Warsaw, IN), cementless	71% (35/49)	100% (49/49)	0% (0/49)
		2D									
Zeng et al. [26]	DDH	3D	20	Not Reported				Pinnacle (DePuy, Warsaw, IN, USA), cementless	70% (14/20)	100% (20/20)	0% (0/20)
		2D	20						25% (5/20)	70% (14/20)	30% (6/20)
Hou et al. [27]	DDH/OA/ON/AS/RA	3D	59	SUMMIT and CORAIL (DePuy Synthes), cementless	76.27% (45/59)	93.22% (55/59)	6.77% (4/59)	PINNACLE acetabular cup (DePuy Synthes)	71.19% (42/59)	93.22% (55/59)	6.77% (4/59)
		2D	59		49.15% (29/59)	74.57% (44/59)	25.42% (15/59)		40.68% (24/59)	77.96% (46/59)	22.03% (13/59)
Anwar et al. [28]	AVN/DDH/FNF/OA	AI-3D	117	Corail, Summit, or Trilock (DePuy Synthes), cementless	65.8% (77/117)			Pinnacle® (DePuy Synthes, Warsaw, IN, US)	66.67% (78/117)		
		2D	117		37.61% (44/117)				30.77% (36/117)		
Ding et al. [31]	AVN/DDH/OA/RA/FNF	AI-3D	316	Tri-Lock (DePuy Synthes), cementless	-	94.3% (298/316)	5.7% (18/316)	PINNACLE cup (DePuy Synthes)	-	97.5% (308/316)	2.5% (8/316)
		2D	316		-	79.1% (250/316)	20.9% (66/316)		-	84.5% (267/316)	15.5% (49/316)
Lu et al. [32]	high hip dislocation	AI-3D	49	Summit or Corail (DePuy Synthes) — cementless	65.31% (32/49)	95.92% (47/49)	4.08% (2/49)	Pinnacle acetabular cups (DePuy Synthes)	59.18% (29/49)	81.63% (40/49)	18.37% (9/49)
		2D	43		41.86% (18/43)	93.02% (40/43)	6.98% (3/43)		30.23% (13/43)	93.02% (40/43)	6.98% (3/43)
Wu et al. [33]	DDH	AI-3D	34	SUMMIT® stem (Johnson & Johnson) — cementless	67.64% (23/34)	97.0% (33/34)	2.94% (1/34)	PINNACLE® cup (Johnson & Johnson)	55.88% (19/34)	82.35% (28/34)	17.64% (6/34)
		2D	27		40.7% (11/27)	88.8% (24/27)	11.1% (3/27)		29.6% (8/27)	92.59% (25/27)	7.4% (2/27)
Zheng et al. [40]	DDH/post-lumbar spine surgery with hip symptoms/post-traumatic hip disease/hip ankylosis/revision for prosthesis failure	AI-3D	29	Johnson & Johnson hip prosthesis specific stem model not specified in paper		82.8% (24/29)	17.2% (5/29)	Johnson & Johnson (specific model not specified in excerpt)		75.9% (22/29)	7.29% (7/29)
		2D	27			55.5% (15/27)	44.5% (12/27)			44.4% (12/27)	55.6% (15/27)
Xie et al. [35]	DDH	AI-3D	164	Not Reported				Pinnacle cup (DePuy, Warsaw, IN, USA)	84.1% (138/164)	95.1% (156/164)	4.9% (8/164)
		2D	164						64.0% (105/164)	81.1% (133/164)	18.9% (31/164)
Yang et al. [36]	FNF/FNA/OA/DDH	AI-3D	220	Tri-Lock (DePuy Orthopaedics), cementless	79.54% (175/220)	95.45% (210/220)	4.55% (10/220)	Pinnacle acetabular cup (DePuy Orthopaedics, USA)	76.81% (169/220)	90.45% (199/220)	9.55% (21/220)
		2D	220		45.90% (101/220)	80.90% (178/220)	19.1% (42/220)		40.45% (89/220)	77.72% (171/220)	22.28% (49/220)
Li et al. [6]	AVN (unilateral)	AI-3D	55	DePuy Synthes prosthesis, specific stem model not specified in paper	87.3% (48/55)	100% (55/55)	0% (0/55)	Not reported	90.9% (50/55)	100% (55/55)	0% (0/55)
		2D	54		66.7% (36/54)	87.03% (47/54)	12.97% (7/54)		72.2% (39/54)	85.45% (47/54)	14.55% (8/54)
Cheng et al. [30]	FNF/FNA/OA	AI-3D	31	Smith and Nephew (SN) femoral stem, specific stem model not specified in paper	83% (26/31)			Smith and Nephew (SN) acetabular cup (sizes 36–58 mm, 2 mm increments)	80.6% (25/31)		
		2D	31		67.74% (21/31)				61.29% (19/31)		
Chen et al. [29]	ON/DDH/OA/old Fx/AS/RA	AI-3D	60	Corail and Trilock (DePuy Orthopaedics), cementless	55% (33/60)	96.67% (58/60)	3.33% (2/60)	Pinnacle Cup (DePuy Orthopaedics, Warsaw, IN, United States)	66.67% (40/60)	96.67% (58/60)	3.33% (2/60)
		2D	60		31.67% (19/60)	65% (39/60)	35% (21/60)		20% (12/60)	55% (33/60)	45% (27/60)
Wu et al. [34]	AVN/DDH/OA/RA/FNF/AS	AI-3D	95	SUMMIT™ handle (Johnson & Johnson), cementless (design not specified in paper)	64.2% (61/95)	97.9% (93/99)	6.06% (6/99)	PINNACLE® cup (Johnson & Johnson)	53.7% (51/95)	91.6% (87/95)	8.4% (8/95)
		2D	66		43.9% (29/66)	84.8% (56/66)	15.15% (10/66)		37.9% (25/66)	87.9% (58/66)	12.12% (8/66)
Zhang et al. [37]	DDH/AVN/FNF/hip arthropathy	AI-3D	19	Corail femoral stem (DePuy), cementless (design not specified in paper)	89.5% (17/19)	94.7% (18/19)	5.3% (1/19)	PINNACLE prosthesis (DePuy)	84.2% (16/19)	100% (19/19)	0% (0/19)
		2D	26		23.1% (6/26)	76.92% (20/26)	23.07% (6/26)		15.4% (4/26)	50% (13/26)	50% (13/26)
Hou et al. [27]	DDH/OA/ON/AS/RA	AI-3D	59	SUMMIT and CORAIL (DePuy Synthes), cementless	71.19% (42/59)	93.22% (55/59)	6.77% (4/59)	PINNACLE acetabular cup (DePuy Synthes)	74.58% (44/59)	94.91% (56/59)	5.08% (3/59)
		2D	59		49.15% (29/59)	74.57% (44/59)	25.42% (15/59)		40.68% (24/59)	77.96% (46/59)	22.03% (13/59)

> ± 2 means an absolute discrepancy of > 2

*DDH* developmental dysplasia of the hip, *ON* osteonecrosis (avascular necrosis), *OA* osteoarthritis, *RDC* rapidly destructive coxarthrosis, *AS* ankylosing spondylitis, *RA* rheumatoid arthritis, *FNF* femoral neck fracture, *N* number, *NR* not reported

### Intraoperative and postoperative outcomes

**Fig. 5:**
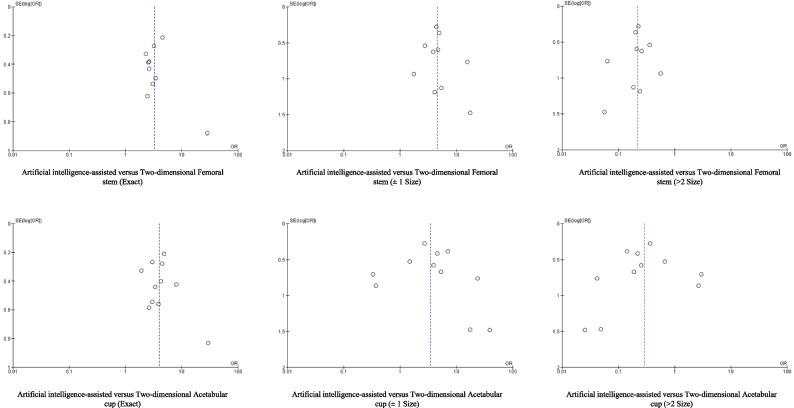
Forest plots comparing artificial intelligence (AI)–assisted versus two-dimensional (2D) methods for functional and postoperative clinical outcomes, including Harris Hip Score, abduction angle, leg length discrepancy (LLD), operative time, and blood loss

Pooled analysis showed that AI-assisted planning was associated with a significant reduction in blood loss compared with 2D planning (MD: − 36.21; 95% CI − 46.58 to − 25.85; *p*-value < 0.001; I^2^ = 0%) (Fig. 5). Furthermore, Operative duration was also significantly shorter in the AI-based approaches (MD: − 15.54; 95% CI − 24.25 to − 6.83; *p*-value = 0.005; I^2^ = 93%) compared to 2D planning (Fig. 5). Leave-one-out analyses showed the reduction in operative time remained statistically significant after the omission of each study. Postoperative LLD was significantly lower with AI-based planning relative to 2D templating (MD: − 1.58; 95% CI − 2.22 to − 0.93; *p*-value < 0.001; I^2^ = 81%) (Fig. 5). The association remained significant in leave-one-out analyses.

Functional status measured by HHS was significantly higher at final follow-ups (MD: 0.73; 95% CI 0.03–1.43; *p*-value = 0.04; I^2^ = 55%) with the AI-assisted planning. However, leave-one-out sensitivity analyses indicated that this finding was not robust, with statistical significance lost after exclusion of certain individual studies (see supplementary material). In addition, postoperative abduction angle was significantly higher in the AI-based templating (MD: 1.64; 95% CI 0.37–2.92; *p*-value = 0.01; I^2^ = 0%), compared with 2D templating. Clinical outcomes are summarized in Table 3.

**Table 3:** Perioperative and postoperative outcomes: AI-assisted planning versus two-dimensional templating

Authors	Planning Method	N	HHS Score		LLD (mm)		Abduction Angle	Intraoperative Blood Loss (ml)	Operative Time(min)
			Pre-Op	Post-Op (Last F/U)	Pre-Op	Post-Op (Last F/U)			
Lu et al. [32]	AI-3D	49	42.1 ± 8.7	85.86 ± 3.93	11.22 ± 6.83	1.21 ± 0.95		228.57 ± 53.97	70.59 ± 11.15
	2D	43	43.5 ± 9.8	84.86 ± 4.51	14.23 ± 8.64	1.85 ± 1.48		251.16 ± 55.94	75.65 ± 10.80
Wu et al. [33]	AI-3D	34	61.01 ± 4.35	86.38 ± 4.04	9.89 ± 8.88	1.64 ± 1.78	37.46° ± 4.53°		78.8 ± 16.9
	2D	27	62.89 ± 6.24	84.37 ± 5.30	14.01 ± 11.18	3.53 ± 3.09	36.28° ± 6.76°		83.4 ± 17.5
Zheng et al. [40]	AI-3D	29	46 ± 1.12	80.59 ± 1.50	9 (5.5–15.85)	6 ± 0.87		140 (130 to 180)	95 (90 to 100)
	2D	27	46 ± 1	80.30 ± 1.96	6 (3–12)	8.5 ± 0.8		160 (150 to 200)	100 (90 to 115)
Yang et al. [36]	AI-3D	220	49.55 ± 3.55	85.51 ± 3.69		0.71 ± 0.54		229.81 ± 97.69	43.22 ± 12.65
	2D	220	49.08 ± 3.09	84.88 ± 2.68		1.14 ± 0.59		267.17 ± 113.88	68.36 ± 14.45
Li et al. [6]	AI-3D	55	67.51 ± 5.31	94.98 ± 2.31		4.05 ± 1.67	42.95 ± 5.70	172.15 ± 38.89	114.24 ± 21.73
	2D	54	63.76 ± 4.73	94.07 ± 2.54		5.94 ± 1.65	41.28 ± 6.85	211.30 ± 49.08	145.72 ± 32.42
Cheng et al. [30]	AI-3D	31	48.1 ± 9.7	81.9 ± 6.5				177.4 ± 45.5	84.2 ± 19.8
	2D	31	49.2 ± 4.7	74.7 ± 11.1				231.0 ± 76.7	100.3 ± 25.9
Chen et al. [36]	AI-3D	60				5.03 ± 3.67			
	2D	60				5.68 ± 4.06			
Wu et al. [34]	AI-3D	95			9.45 ± 7.78	2.18 ± 2.70	36.85° ± 4.82°		78.5 ± 18.5
	2D	66			11.06 ± 9.26	4.42 ± 3.85	35.06° ± 6.07°		83.6 ± 18.7
Zhang et al. [38]	AI-3D	44				3.8 ± 1.92			77 (63 to 89)
	2D	40				4.6 ± 2.25			68(57 to 83)
Zhang et al. [37]	AI-3D	19						323.68 ± 222.59	104.32 ± 18.10
	2D	26						376.92 ± 247.08	134.34 ± 6.19
Zheng et al. [39]	AI-3D	30	56.2 ± 15.9	95.5 ± 3.1		4.5 ± 2.0		405.7 ± 176.6	79.7 ± 20.7
	2D	30	54.6 ± 13.4	96.0 ± 2.0		6.4 ± 2.9		397 ± 188.3	87.0 ± 26.7

*LLD* leg length discrepancy, *HHS* Harris Hip Score, *N* number of cases

## Discussion

Preoperative planning in total joint arthroplasty is essential for achieving accurate implant sizing, restoring biomechanics, and optimizing surgical and mechanical outcomes. In our pooled analysis, 3D planning was associated with higher accuracy of femoral stem and acetabular cup size prediction than conventional 2D templating, including higher exact size prediction and prediction within one size, and fewer major sizing deviations of more than two sizes. Artificial intelligence-assisted planning not only significantly improved stem and cup sizing accuracy relative to 2D but also translated into better intraoperative and postoperative outcomes, including reduced blood loss, shorter operative time, lower LLD, and higher abduction angle.

Three-dimensional and AI-assisted planning showed consistently improved accuracy compared to 2D planning across femoral stem sizing metrics. Unlike total knee arthroplasty (TKA), where pooled evidence from 16 studies, including 1,189 TKAs, reported that 2D digital templating predicts femoral component size within one size in approximately 96% of cases, the incremental benefit of advanced planning appears more pronounced in THA, supporting higher implementation value [41]. In three CT-based 3D templating studies using Hip-Plan and Hip-Op software, assessment of the femoral canal and cortical envelope, together with offset and limb length, improved the match between planned and implanted stem size and provided an upper safety limit for stem diameter and length to avoid cortical breach [3, 22, 23]. Accurate stem sizing also facilitates better control of stem anteversion and reconstruction of the hip centre of rotation, particularly in complex femoral morphologies such as deformed femurs and severe developmental dysplasia of the hip (DDH) [18]. These potential benefits may be most relevant for short bone-preserving stems and cementless stems, where press-fit and sizing errors are closely linked to early complications [1, 2].

More accurate socket planning by advanced planning approaches may support restoration of acetabular component anteversion and reduce the risk of wear, impingement, and dislocation, particularly when native acetabular landmarks are poorly visualized on radiographs [7, 22, 23]. Improved predictability of cup size can simplify instrumentation by reducing trial-and-error reaming and unplanned size changes [1]. In dysplastic hips, advanced planning may improve bone coverage by characterizing the type and degree of acetabular dysplasia and guiding cup position in shallow or steep domes [18, 26]. Accurate cup placement may also be valuable in revision THA with acetabular defects, where normal bony landmarks are often lost, and model-based planning can help optimize host bone contact and augment configuration [16]. Moreover, accurate 3D cup sizing may limit offset changes that predispose to instability or trochanteric pain in direct anterior approach (DAA) THA, and may be critical for achieving a stable, screwless press-fit while preserving the anterior and posterior acetabular walls and maintaining bone stock for future revision [4, 21].

In this analysis, AI-assisted planning showed significantly higher accuracy in femoral stem and cup sizing across all metrics. It may particularly support less-experienced surgeons by providing rapid, detailed 3D planning without complex manual workflows, with reduced planning time, manpower, and intraoperative fluoroscopy [29]. These efficiencies may be particularly relevant in routine cases and busy centers [34, 36]. Regarding functional outcomes, postoperative HHS was statistically higher with AI-assisted planning, but the mean difference of less than 1 point is well below the established minimal clinically important improvement of 15.9–18 points and was not robust in leave-one-out analysis [42, 43]. This finding is therefore statistically significant but clinically limited.

AI-assisted planning reduced operative time by about 16 min, likely reflecting a more streamlined workflow, as accurate preoperative sizing reduces the number of reaming and broaching attempts and trial component fittings required intraoperatively [6, 32, 36, 37]. The 36 mL reduction in intraoperative blood loss follows from the same mechanism, as fewer instrumentation passes mean less soft tissue trauma [30, 39]. LLD was reduced by about 1.6 mm, consistent with patient-specific 3D templating of neck cut, offset, and head–neck combination, rather than magnification-prone 2D landmarks [29, 33, 36, 37, 39]. The 1.6° higher mean cup abduction is clinically small, but two contributing studies reported higher Lewinnek and Callanan safe-zone placement with AI-assisted planning, indicating fewer outliers [33, 34]. This gain without intraoperative navigation likely reflects planning-guided manual execution, where a patient-specific 3D plan is translated intraoperatively using anatomical landmarks such as the transverse acetabular ligament and residual labrum [44].

All included AI studies were conducted in Chinese centers, mostly using one platform. Chinese THA cohorts may differ from Western cohorts in ways directly relevant to implant sizing and outcomes; for instance, DDH prevalence appears relatively more common in Chinese adults than US adults (1.5% vs 0.1%), and Asian femurs show smaller medial offset and wider isthmus diameter and lower BMI (22.9 vs. 26.3) than Caucasians, predisposing to fit-and-fill mismatch with stems designed from Caucasian data [45–48]. These differences mean our AI accuracy estimates require external validation in Western cohorts and non-AIHIP platforms before clinical extrapolation.

This study had several limitations. Most included studies were retrospective cohorts; therefore, confounding factors, such as surgeon experience and case complexity, may have influenced the observed differences and increased the risk of bias. There was substantial heterogeneity. Potential small-study effects were detected for the AI-assisted major deviation outcomes for both femoral stem and acetabular cup sizing, suggesting that the magnitude of benefit may be uncertain and should be interpreted with caution. Postoperative functional status was evaluated at heterogeneous time points (one to 24 months), which increased variability and should be considered when interpreting the result. Follow-up was reported inconsistently, and when reported, it was most commonly within 24 months; therefore, the effect of improved planning on long-term complications and implant survival remains uncertain. Moreover, none of the included studies directly compared AI-assisted planning to conventional 3D planning, and few reported data on costs, additional radiation exposure, or learning curves. Nonetheless, this review had important strengths; it evaluated both 3D and AI planning separately compared with 2D planning, and assessed not only component sizing accuracy but also its association with clinically relevant outcomes. Finally, all studies evaluating AI-assisted planning were conducted in China, and most used the AIHIP platform, limiting external validity. Patient anatomy and characteristics, DDH prevalence and severity, access to technology, and surgical techniques may differ across healthcare systems. Therefore, future studies should validate these results across diverse populations and AI algorithms, directly compare AI-assisted and conventional 3D workflows, and determine whether improvements in implant sizing accuracy translate into better long-term outcomes, including complications and implant survival.

## Conclusion

Advanced preoperative planning improved implant sizing accuracy, including both femoral stem and acetabular cup sizing, compared with 2D templating in THA using multiple accuracy criteria. More favorable intraoperative and early postoperative outcomes, such as reduced blood loss, lower LLD, and shorter operative time, were associated with AI-assisted approaches. Given heterogeneity across some endpoints, limited long-term follow-up, and the geographic and software concentration of AI-assisted studies, standardized multicenter studies are needed to validate these findings across diverse patient populations, implant systems, and AI algorithms.

## Supplementary Information

Supplementary Material 1.

## Data Availability

The datasets used and/or analysed during the current study are available from the corresponding author on reasonable request.
