# Supplementary material for: Three-dimensional and artificial intelligence-assisted preoperative templating in total hip arthroplasty: a systematic review and meta-analysis
Source: Arthroplasty. 2026 Aug 4;8:56. doi: 10.1186/s42836-026-00417-3 (PMC13435940; doi:10.1186/s42836-026-00417-3)
Supplement: Supplementary file 1 [file 42836_2026_417_MOESM1_ESM.docx]

**Supplementary material**

**Supplementary Table S1.** PICO framework for study selection

| **Component** | **Description** |
| --- | --- |
| Population | Adult patients (≥18 years) undergoing total hip arthroplasty (THA) across elective orthopedic settings. |
| Intervention | Preoperative planning using three-dimensional (3D) planning or artificial intelligence (AI)–assisted planning, including CT-based or model-based planning systems. |
| Comparison | Conventional two-dimensional (2D) radiographic templating for preoperative planning. |
| Outcomes | Primary outcomes: Femoral stem and acetabular cup sizing accuracy (exact size agreement, agreement within ±1 size, ≥2-size deviation). Secondary outcomes: Intraoperative and postoperative measures, including operative time, blood loss, leg length discrepancy (LLD), Harris Hip Score (HHS), abduction angle, and other relevant clinical or surgical outcomes. |
| Study Design | Systematic review and meta-analysis |

**Supplementary Table S2.** Systematic search strategy protocol

| **Database** | **Search Query** | | **Results** |
| --- | --- | --- | --- |
| PubMed | ("arthroplasty, replacement, hip"[MeSH Terms] OR "arthroplasty replacement hip*"[Title/Abstract] OR "acetabulum/surgery"[MeSH Terms] OR "acetabulum surgery*"[Title/Abstract] OR "arthroplasty, replacement, hip/methods"[MeSH Terms] OR "arthroplasty replacement hip methods*"[Title/Abstract] OR "osteoarthritis, hip/surgery"[MeSH Terms] OR (("Osteoarthritis"[MeSH Terms] OR "Osteoarthritis"[All Fields] OR "osteoarthritides"[All Fields]) AND "hip surgery*"[Title/Abstract]) OR "osteoarthritis, hip/etiology"[MeSH Terms] OR "osteoarthritis hip etiology*"[Title/Abstract] OR "developmental dysplasia of the hip/surgery"[MeSH Terms] OR ((("developmental"[All Fields] OR "developmentally"[All Fields]) AND ("dysplasia"[All Fields] OR "dysplasias"[All Fields])) AND "hip surgery*"[Title/Abstract]) OR "developmental dysplasia of the hip/complications"[MeSH Terms] OR ((("developmental"[All Fields] OR "developmentally"[All Fields]) AND ("dysplasia"[All Fields] OR "dysplasias"[All Fields])) AND "hip complications*"[Title/Abstract])) AND ("printing, three dimensional"[MeSH Terms] OR "printing three dimensional*"[Title/Abstract] OR "Prostheses and Implants"[MeSH Terms] OR "prostheses and implants*"[Title/Abstract] OR "Hip Prosthesis"[MeSH Terms] OR "hip prosthesis*"[Title/Abstract] OR "Titanium"[MeSH Terms] OR "titanium*"[Title/Abstract] OR "prosthesis fitting/instrumentation"[MeSH Terms] OR (("prosthesis fitting"[MeSH Terms] OR ("prosthesis"[All Fields] AND "Fitting"[All Fields]) OR "prosthesis fitting"[All Fields]) AND "instrumentation*"[Title/Abstract]) OR "prosthesis fitting/methods"[MeSH Terms] OR (("Prostheses and Implants"[MeSH Terms] OR ("Prostheses"[All Fields] AND "implants"[All Fields]) OR "Prostheses and Implants"[All Fields] OR "prosthesis"[All Fields]) AND "fitting methods*"[Title/Abstract]) OR "surgery, computer assisted/instrumentation"[MeSH Terms] OR (("Surgery"[MeSH Subheading] OR "Surgery"[All Fields] OR "surgical procedures, operative"[MeSH Terms] OR ("surgical"[All Fields] AND "procedures"[All Fields] AND "operative"[All Fields]) OR "operative surgical procedures"[All Fields] OR "general surgery"[MeSH Terms] OR ("general"[All Fields] AND "Surgery"[All Fields]) OR "general surgery"[All Fields] OR "surgery s"[All Fields] OR "surgerys"[All Fields] OR "surgeries"[All Fields]) AND "computer assisted instrumentation*"[Title/Abstract]) OR "surgery, computer assisted/methods"[MeSH Terms] OR "surgery computer assisted methods*"[Title/Abstract] OR "Patient-Specific Modeling"[MeSH Terms] OR "patient specific modeling*"[Title/Abstract]) AND ("Preoperative Period"[MeSH Terms] OR "preoperative period*"[Title/Abstract] OR "Preoperative Care"[MeSH Terms] OR "preoperative care*"[Title/Abstract] OR "preoperative care/methods"[MeSH Terms] OR "preoperative care methods*"[Title/Abstract] OR "Patient Care Planning"[MeSH Terms] OR "patient care planning*"[Title/Abstract]) | | 344 |
| Web of  Science | TS=("arthroplasty replacement hip*" OR "acetabulum surgery*" OR "arthroplasty replacement hip methods*"  OR ("osteoarthritis" AND "hip surgery*")  OR "osteoarthritis hip etiology*"  OR (("developmental" OR "developmentally") AND ("dysplasia" OR "dysplasias") AND "hip surgery*")  OR (("developmental" OR "developmentally") AND ("dysplasia" OR "dysplasias") AND "hip complications*"))  AND  TS=("printing three dimensional*" OR "prostheses and implants*" OR "hip prosthesis*" OR "titanium*"  OR ("prosthesis fitting" AND "instrumentation*")  OR ("prostheses and implants" AND "fitting methods*")  OR ("surgery" AND "computer assisted instrumentation*")  OR "surgery computer assisted methods*"  OR "patient specific modeling*")  AND  TS=("preoperative period*" OR "preoperative care*" OR "preoperative care methods*" OR "patient care planning*") | 589 | |
| Scopus | (TITLE-ABS-KEY("arthroplasty replacement hip*") OR TITLE-ABS-KEY("acetabulum surgery*") OR TITLE-ABS-KEY("arthroplasty replacement hip methods*") OR (TITLE-ABS-KEY("osteoarthritis") AND TITLE-ABS-KEY("hip surgery*")) OR TITLE-ABS-KEY("osteoarthritis hip etiology*") OR ((TITLE-ABS-KEY("developmental") OR TITLE-ABS-KEY("developmentally")) AND (TITLE-ABS-KEY("dysplasia") OR TITLE-ABS-KEY("dysplasias")) AND TITLE-ABS-KEY("hip surgery*")) OR ((TITLE-ABS-KEY("developmental") OR TITLE-ABS-KEY("developmentally")) AND (TITLE-ABS-KEY("dysplasia") OR TITLE-ABS-KEY("dysplasias")) AND TITLE-ABS-KEY("hip complications*")))AND  (TITLE-ABS-KEY("printing three dimensional*") OR TITLE-ABS-KEY("prostheses and implants*") OR TITLE-ABS-KEY("hip prosthesis*") OR TITLE-ABS-KEY("titanium*") OR (TITLE-ABS-KEY("prosthesis fitting") AND TITLE-ABS-KEY("instrumentation*")) OR (TITLE-ABS-KEY("prostheses and implants") AND TITLE-ABS-KEY("fitting methods*")) OR (TITLE-ABS-KEY("surgery") AND TITLE-ABS-KEY("computer assisted instrumentation*")) OR TITLE-ABS-KEY("surgery computer assisted methods*") OR TITLE-ABS-KEY("patient specific modeling*")) AND (TITLE-ABS-KEY("preoperative period*") OR TITLE-ABS-KEY("preoperative care*") OR TITLE-ABS-KEY("preoperative care methods*") OR TITLE-ABS-KEY("patient care planning*")) | 1120 | |

**Supplementary file S3. JBI quality assessment result: Cohort studies**

Q1. Were the two groups similar and recruited from the same population?
Q2. Were the exposures measured similarly to assign people to both the exposed and unexposed groups?
Q3. Was the exposure measured in a valid and reliable way?
Q4. Were confounding factors identified?
Q5. Were strategies to deal with confounding factors stated?
Q6. Were the groups/participants free of the outcome at the start of the study (or at the moment of exposure)?
Q7. Were the outcomes measured in a valid and reliable way?
Q8. Was the follow-up time reported and sufficient for outcomes to occur?
Q9. Was follow-up complete and, if not, were the reasons for loss to follow-up described and explored?
Q10. Were strategies to address incomplete follow-up utilized?
Q11. Was an appropriate statistical analysis used?

Y: yes, N: no, U: unclear, NA: not applicable

| Cohort study | Q1 | Q2 | Q3 | Q4 | Q5 | Q6 | Q7 | Q8 | Q9 | Q10 | Q11 |
| --- | --- | --- | --- | --- | --- | --- | --- | --- | --- | --- | --- |
| Tostain et al. | Y | Y | Y | Y | N | Y | Y | Y | Y | Y | Y |
| Vaquinhas et al. | Y | Y | Y | Y | Y | Y | Y | Y | U | N | Y |
| Aubert et al. | Y | Y | Y | Y | Y | Y | Y | Y | Y | NA | Y |
| Zeng et al. | NA | NA | Y | NA | NA | Y | Y | U | Y | NA | Y |
| Anwar et al. | NA | NA | Y | NA | NA | Y | Y | Y | Y | NA | Y |
| Ding et al. | NA | NA | Y | NA | NA | Y | Y | Y | U | N | Y |
| Lu et al. | Y | Y | Y | Y | N | Y | Y | Y | Y | N | Y |
| Wu et al. | Y | Y | Y | Y | Y | Y | Y | Y | Y | NA | Y |
| Zheng et al. | Y | Y | Y | Y | Y | Y | Y | Y | Y | N | Y |
| Xie et al. | NA | NA | U | NA | NA | Y | Y | Y | Y | NA | Y |
| Yang et al. | Y | Y | Y | Y | Y | Y | Y | U | U | N | Y |
| Li et al. | Y | Y | Y | Y | N | Y | Y | Y | Y | NA | Y |
| Cheng et al. | Y | Y | Y | Y | Y | Y | U | Y | Y | NA | Y |
| Chen et al. | Y | Y | U | Y | N | Y | Y | Y | U | N | Y |
| Wu et al. | Y | Y | Y | Y | N | Y | U | Y | Y | NA | Y |
| Zhang et al. | Y | Y | Y | Y | N | Y | Y | Y | Y | N | Y |
| Ogawa et al. | NA | NA | Y | NA | NA | Y | Y | Y | Y | N | Y |

**Supplementary file S4. JBI quality assessment result: randomized controlled studies**

Q1. Was true randomization used for assignment of participants to treatment groups?
Q2. Was allocation to treatment groups concealed?
Q3. Were treatment groups similar at baseline?
Q4. Were participants blinded to treatment assignment?
Q5. Were those delivering the treatment blinded to treatment assignment?
Q6. Were treatment groups treated identically other than the intervention of interest?
Q7. Were outcome assessors blinded to treatment assignment?
Q8. Were outcomes measured in the same way for treatment groups?
Q9. Were outcomes measured in a reliable way?
Q10. Was follow-up complete and, if not, were between-group differences in follow-up described and analyzed?
Q11. Were participants analyzed in the groups to which they were randomized?
Q12. Was appropriate statistical analysis used?
Q13. Was the trial design appropriate, and were any deviations from standard RCT design accounted for?

Y: yes, N: no, U: unclear, NA: not applicable

| RCT | Q1 | Q2 | Q3 | Q4 | Q5 | Q6 | Q7 | Q8 | Q9 | Q10 | Q11 | Q12 | Q13 |
| --- | --- | --- | --- | --- | --- | --- | --- | --- | --- | --- | --- | --- | --- |
| Brenneis et al. | Y | U | Y | N | N | Y | Y | Y | Y | N | N | Y | Y |
| Fontalis et al. | Y | Y | Y | NA | N | Y | Y | Y | Y | Y | Y | Y | Y |
| Sariali et al. | Y | U | Y | N | N | N | N | N | U | Y | Y | Y | N |
| Zhang et al. | Y | Y | Y | U | N | Y | Y | Y | Y | Y | U | Y | Y |
| Zheng et al. | N | N | Y | N | N | Y | U | Y | U | U | N | Y | N |

**Supplementary file S5. JBI quality assessment result: cross-sectional studies.**

Q1. Were the criteria for inclusion in the sample clearly defined?
Q2. Were the study subjects and the setting described in detail?
Q3. Was the exposure measured in a valid and reliable way?
Q4. Were objective, standard criteria used for measurement of the condition?
Q5. Were confounding factors identified?
Q6. Were strategies to deal with confounding factors stated?
Q7. Were the outcomes measured in a valid and reliable way?
Q8. Was appropriate statistical analysis used?

Y: yes, N: no, U: unclear, NA: not applicable

| Cross-sectional study | Q1 | Q2 | Q3 | Q4 | Q5 | Q6 | Q7 | Q8 |
| --- | --- | --- | --- | --- | --- | --- | --- | --- |
| Schiffner et al. | Y | Y | Y | Y | Y | Y | Y | Y |
| Crutcher et al. | Y | Y | Y | Y | Y | Y | Y | Y |
| Viceconti et al. | Y | Y | Y | Y | Y | Y | Y | Y |
| Hou et al. | Y | Y | Y | Y | Y | Y | Y | Y |

**Supplementary file S6. JBI quality assessment result: case-series studies.**

Q1. Were there clear criteria for inclusion in the case series?
Q2. Was the condition measured in a standard, reliable way for all participants?
Q3. Were valid methods used for identification of the condition for all participants?
Q4. Did the case series have consecutive inclusion of participants?
Q5. Did the case series have complete inclusion of participants?
Q6. Was there clear reporting of participant demographics?
Q7. Was there clear reporting of clinical information of participants?
Q8. Were outcomes or follow-up results clearly reported?
Q9. Was there clear reporting of presenting site(s)/clinic(s) demographic information?
Q10. Was statistical analysis appropriate?

Y: yes, N: no, U: unclear, NA: not applicable

| Case series | Q1 | Q2 | Q3 | Q4 | Q5 | Q6 | Q7 | Q8 | Q9 | Q10 |
| --- | --- | --- | --- | --- | --- | --- | --- | --- | --- | --- |
| Knafo et al. | Y | Y | Y | Y | Y | Y | Y | Y | Y | Y |
| Hassani et al. | Y | Y | Y | Y | Y | Y | Y | Y | Y | Y |
| Inoue et al. | Y | Y | Y | Y | Y | Y | Y | Y | Y | Y |
| Sariali et al. | Y | Y | Y | Y | Y | Y | Y | Y | Y | Y |
| Wu et al. | Y | Y | Y | Y | Y | Y | Y | Y | Y | Y |

**Supplementary file S7. JBI quality assessment result: case-control study.**

Q1. Were the groups comparable other than the presence of disease in cases or the absence of disease in controls?
Q2. Were cases and controls matched appropriately?
Q3. Were the same criteria used for identification of cases and controls?
Q4. Was exposure measured in a standard, valid, and reliable way?
Q5. Was exposure measured in the same way for cases and controls?
Q6. Were confounding factors identified?
Q7. Were strategies to deal with confounding factors stated?
Q8. Were outcomes assessed in a standard, valid, and reliable way for cases and controls?
Q9. Was the exposure period of interest long enough to be meaningful?
Q10. Was appropriate statistical analysis used?

Y: yes, N: no, U: unclear, NA: not applicable

| Case-control study | Q1 | Q2 | Q3 | Q4 | Q5 | Q6 | Q7 | Q8 | Q9 | Q10 |
| --- | --- | --- | --- | --- | --- | --- | --- | --- | --- | --- |
| Mainard et al. | NA | NA | Y | Y | Y | N | N | Y | Y | Y |

**Supplementary Table S8. Leave-one-out sensitivity analysis**

| **Outcome** | **Omitted study** | **Pooled effect** | **95% CI** | **P-value upon removal** | **I² (%)** |
| --- | --- | --- | --- | --- | --- |
| HHS |  |  |  |  |  |
|  | Zheng et al. 2025 | 0.89 | 0.01 to 1.78 | 0.03 | 60 |
|  | Zheng et al. 2024 | 0.92 | 0.20 to 1.64 | 0.07 | 51 |
|  | Yang et al. 2023 | 0.87 | -0.12 to 1.87 | 0.09 | 62 |
|  | Wu et al. 2023 | 0.64 | -0.09 to 1.47 | 0.09 | 58 |
|  | Lu et al. 2025 | 0.72 | -0.07 to 1.51 | 0.08 | 62 |
|  | Li et al. 2025 | 0.75 | -0.15 to 1.64 | 0.1 | 61 |
|  | Cheng et al. 2024 | 0.57 | 0.17 to 0.98 | < 0.001 | 1 |
| LLD |  |  |  |  |  |
|  | Chen et al. 2022 | -1.68 | -2.36 to -0.99 | <0.001 | 83 |
|  | Li et al. 2025 | -1.52 | -2.30 to -0.75 | <0.001 | 83 |
|  | Lu et al. 2025 | -1.79 | -2.33 to -1.24 | <0.001 | 62 |
|  | Wu et al. 2023 | -1.54 | -2.25 to -0.84 | <0.001 | 84 |
|  | Wu et al. 2023 | -1.49 | -2.20 to -0.78 | <0.001 | 83 |
|  | Zhang et al. 2023 | -1.69 | -2.39 to -1.00 | <0.001 | 82 |
|  | Zheng et al. 2024 | -1.54 | -2.25 to -0.83 | <0.001 | 84 |
|  | Zheng et al. 2025 | -1.39 | -1.96 to -0.82 | <0.001 | 63 |
| Operative time |  |  |  |  |  |
|  | Cheng et al. 2024 | -15.47 | -25.00 to -5.95 | 0.001 | 94 |
|  | Li et al. 2025 | -13.41 | -22.74 to -4.08 | 0.005 | 94 |
|  | Lu et al. 2025 | -17.2 | -26.08 to -8.33 | <0.001 | 91 |
|  | Wu et al. 2023 | -17.09 | -26.40 to -7.77 | <0.001 | 94 |
|  | Wu et al. 2023 | -17.14 | -26.43 to -7.85 | <0.001 | 93 |
|  | Yang et al. 2023 | -13.91 | -22.37 to -5.46 | 0.001 | 87 |
|  | Zhang et al. 2024 | -13.48 | -22.94 to -4.02 | 0.005 | 94 |
|  | Zheng et al. 2024 | -16.58 | -25.93 to -7.22 | <0.001 | 94 |
| Cup Size (3D vs 2D) |  |  |  |  |  |
|  | Aubert et al. 2023 | 4.59 | 1.55 to 15.78 | 0.007 | 91 |
|  | Brenneis et al. 2020 | 7.18 | 2.45 to 21.01 | <0.001 | 90 |
|  | Crutcher et al. 2023 | 3.15 | 1.51 to 6.57 | 0.002 | 79 |
|  | Fontalis et al. 2024 | 5.75 | 1.69 to 19.58 | 0.005 | 91 |
|  | Huo et al. 2021 | 5.88 | 1.74 to 20.09 | 0.005 | 91 |
|  | Mainard et al. 2017 | 6.63 | 2.13 to 20.61 | 0.001 | 91 |
|  | Sariali et al. 2012 | 4.38 | 1.54 to 12.42 | 0.006 | 90 |
|  | Schiffner et al. 2018 | 6.58 | 1.95 to 22.23 | 0.002 | 90 |
|  | Zeng et al. 2014 | 5.22 | 1.68 to 16.17 | 0.004 | 91 |

3D: three-dimensional, 2D: two-dimensional, CI: confidence interval, HHS: Harris Hip Score, LLD: leg length discrepancy

**Supplementary Table S9. Egger’s Test analysis**

| **Outcome** | **Number of studies** | **Egger’s Intercept (Bias)** | ***p*-value** | **Conclusion** |
| --- | --- | --- | --- | --- |
| AI vs 2D Femoral stem (Exact) | 10 | 0.13 | 0.89 | No significant bias |
| AI vs 2D Femoral stem (±1 size) | 10 | –1.69 | 0.36 | No significant bias |
| AI vs 2D Femoral stem (>2 sizes) | 10 | –3.88 | 0.002 | Significant bias |
| AI vs 2D Acetabular cup size (Exact) | 11 | 0.85 | 0.51 | No significant bias |
| AI vs 2D Acetabular cup size (±1 size) | 11 | 0.02 | 0.98 | No significant bias |
| AI vs 2D Acetabular cup size (>2 sizes) | 11 | −3.88 | 0.002 | Significant bias |
